# Chiroptical Measurement of Chiral Aggregates at Liquid-Liquid Interface in Centrifugal Liquid Membrane Cell by Mueller Matrix and Conventional Circular Dichroism Methods

**DOI:** 10.3390/molecules16053636

**Published:** 2011-04-29

**Authors:** Hideaki Takechi, Oriol Arteaga, Josep M. Ribo, Hitoshi Watarai

**Affiliations:** 1 Department of Chemistry, Graduate School of Science, Osaka University. 1-1 Machikaneyama-machi, Toyonaka, Osaka, 560-0043, Japan; Email: takechi@chem.sci.osaka-u.ac.jp (H.T.); 2 Department of Chemistry, New York University, 31 Washington Place, Brown Building, 6th floor, New York, NY 1003, USA; Email: oarteaga@ub.edu (O.A.); 3 Department of Organic Chemistry, Institute of Cosmos Science, University of Barcelona. Martí i Franquès 1, 08028 Barcelona, Catalonia, Spain; Email: jmribo@ub.edu (J.M.R.); 4 Institute for NanoScience Design, Osaka University, 1-3 Machikaneyama-machi, Toyonaka, Osaka 560-8531, Japan

**Keywords:** Mueller matrix, liquid-liquid interface, chirality, circular dichroism, linear dichroism, circular birefringence, linear birefringence, porphyrin, aggregation

## Abstract

The centrifugal liquid membrane (CLM) cell has been utilized for chiroptical studies of liquid-liquid interfaces with a conventional circular dichroism (CD) spectropolarimeter. These studies required the characterization of optical properties of the rotating cylindrical CLM glass cell, which was used under the high speed rotation. In the present study, we have measured the circular and linear dichroism (CD and LD) spectra and the circular and linear birefringence (CB and LB) spectra of the CLM cell itself as well as those of porphyrine aggregates formed at the liquid-liquid interface in the CLM cell, applying Mueller matrix measurement method. From the results, it was confirmed that the CLM-CD spectra of the interfacial porphyrin aggregates observed by a conventional CD spectropolarimeter should be correct irrespective of LD and LB signals in the CLM cell.

## 1. Introduction

Circular dichroism (CD) spectra measurements are now widely employed for characterizing and quantifying natural and synthetic chiral compounds. Using a conventional circular dichroism spectropolarimeter, CD spectra have been measured not only for the isotropic samples, but also for various anisotropic samples such as solid, liquid crystal, film, membrane, micelles and gel. However, CD spectra of anisotropic samples are often accompanied by artifacts due to linear dichroism (LD) and linear birefringence (LB). Therefore, it is necessary to pay a lot of care to such chiral artifacts in CD measurements, unless the samples are completely random-orientation systems such as solutions [1,2]. Recently, chiroptical measurements of molecular aggregates at the liquid-liquid interface have been examined by using the centrifugal liquid membrane (CLM) cell, which could generate a very thin oil/water two-phase system with a high specific interfacial area inside the wall of a rotating cylindrical glass cell [3]. However, it was pointed out that there was possibility of inclusion of artifacts in the CLM-CD spectra in some highly oriented molecular aggregate systems [4]. A commercially available instrument for CD measurements may have a high sensitivity to CD signals, but is not designed to measure other optical polarization properties.

Optical measurements of the polarization properties of a sample can be performed by analysis of its Mueller matrix. Recently, transmission Mueller matrix ellipsometry, sometimes referred as Mueller polarimetry, has been applied to the study of anisotropic samples containing oriented molecules [5] or crystals [6,7,8]. Some years ago, the generalized Mueller matrix ellipsometry was applied to anisotropic samples, that could not be handled with a conventional ellipsometry, by the idea that the rigorous polarimetric measurements of optical activity of anisotropic samples should be done by the Mueller matrix measurements. However, it was only recent that this need has been experimentally accomplished [5,9], and the application of Mueller matrix measurement to chiral anisotropic samples is still rare. The difficulty lies in the fact that the magnitude of CD and circular birefringence (CB) signals is usually much smaller than that of absorbance, LD and LB. LD and LB usually hinder the small contributions of CD and CB. In the present study, we have measured all optical property of the CLM cell and chiral porphyrine aggregates formed at the liquid-liquid interface in the CLM cell by Mueller matrix method in order to confirm the applicability of CLM cell for CD measurements of the liquid-liquid interfaces. This is the first report of Mueller matrix measurements of the liquid-liquid interface.

## 2. Theoretical Background

The most comprehensive way to depict the changes in light polarization, as it interacts with a sample, is to use the Stokes-Mueller formalism [10]. The Stokes vector is a 4-element vector with real components, which can represent any polarization state of light, and the Mueller matrix is a 4 × 4 real matrix that describes the changes in light polarization as it interacts with an optical element. Once determined for a sample, this Mueller matrix will allow representing all of the sample’s polarization properties. In addition, any depolarization of the light beam from the sample will be reflected in the Mueller matrix of the sample. The partial or complete determination of the Mueller matrix can be achieved by several different types of polarimeter setups, however, the partial determination is usually not enough for anisotropic samples, since in this case the Mueller matrix elements are not directly related to individual polarization properties of the sample. This relationship is not straightforward; for example, if a compound is chiral and linearly anisotropic, there is not a single element of the Mueller matrix that can be attributed to CD or CB [11,12]. Measurements of CD and CB of liquid isotropic organic compounds are much simpler, since all the linear anisotropies vanish and only pure circular contributions remain.

In the present work, we will use the Stokes–Mueller description of polarized light propagation to specify the irradiance that reaches the detector of the polarimeter. We represent the Stokes vector as (*s_0_*, *s_1_*, *s_2_*, *s_3_*), where the components are the Stokes parameters. The order of the parameters is total intensity, x- or y-axis linear polarization, 45° linear polarization and circular polarization [11].

For thin specimens that show small mixed anisotropies and that CD and CB signals are smaller than LD and LB signals, we can ignore second-order terms in the chiral anisotropies as well as quadratic terms which involve both, linear and chiral anisotropies. In this case the general Mueller matrix of the sample reduces to the following equation [11]:

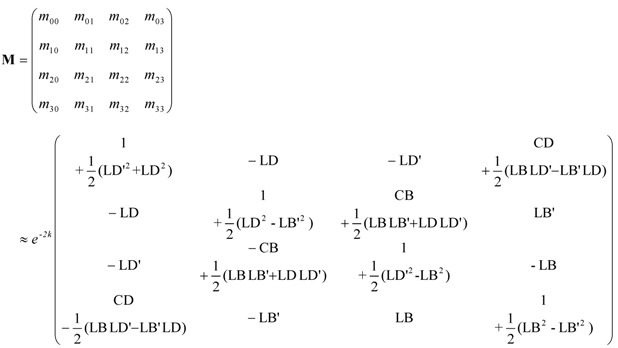
(1)

Here LD’ and LB’ are respectively 45° linear dichroism and 45° linear birefringence [11]. The quantities CD, CB, LD, LB, LD’ and LB’ have the units of radians and are defined in Table 1 [1,11,13,14]. 

**Table 1:** Notations and conventions in anisotropic spectroscopy [1,11,13,14].

Effect	Phenomenological Symbol	Definition	Relation to experiment
Isotropic amplitude absorption	*k*	2*πκl*/*λ_0_*	
Isotropic phase retardation	*η*	2*πnl*/*λ_0_*	
Circular dichroism	CD	*ln* 10(*A_−_* - *A_+_*)/2	*ln* 10*Δε_±_cl*/2
Circular birefringence	CB	2*π*(*n_−_*- *n_+_*)*l*/*λ_0_*	*πα*/90°
(x-y) linear dichroism	LD	*ln* 10(*A_x_* - *A_y_*)/2	*ln* 10*Δε'cl*/2
(x-y) linear birefringence	LB	2*π*(*n_x_* - *n_y_*)*l*/*λ_0_*	2*π*Δ*n'l*/*λ_0_*
45° linear dichroism	LD'	*ln* 10(*A_45_* - *A_135_*)/2	*ln* 10*Δε''cl*/2
45° linear birefringence	LB'	2*π*(*n_45_* - *n_135_*)*l*/*λ_0_*	2*π*Δ*n''*/*λ_0_*

*A* stands for standard absorbance, *n* for refractive index, *l* for pathlength through the medium, *c* for molar concentration, *κ* for the extinction coefficient, *α* for the optical rotation and *λ_o_* for the vacuum wavelength of light. Subscripts specify the polarization of light as x, y, 45° to the x-axis, 135° to the x-axis, circular right (+), or left (−).

The general interpretation of a transmission Mueller matrix for a longitudinally homogeneous medium (regardless of the magnitude of the anisotropies) can be analytically done according to the approach given in Refs. [14,15]. Representative abbreviations used in the present study are listed in Table 2.

**Table 2:** Representative abbreviations used in the present study.

Abbreviation	Full name	Sort
CD	Circular dichroism	Optical property
CB	Circular birefringence	Optical property
LD	Linear dichroism	Optical property
LB	Linear birefringence	Optical property
TPyP	Tetra(4-pyridyl)porphine	Reagent
CuTPPS	Cu(II) tetra(4-sulfonatophenyl)porphine	Reagent
CLM	Centrifugal liquid membrane	Measurement method
2-MGE	Two-modulator generalized ellipsometer	Instrument

## 3. Results and Discussion

### 3.1. Mueller Matrices of CLM Cell

**Figure 1:**
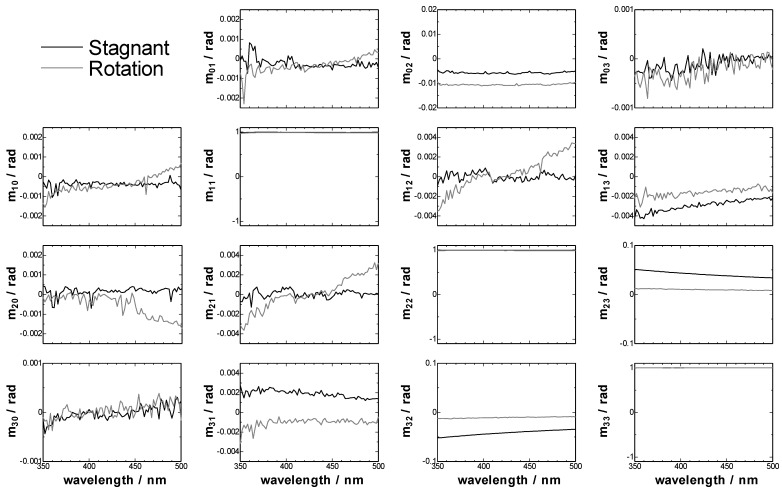
Detail of the observed normalized Mueller matrices of CLM cell.

First we measured the linear birefringence of the empty CLM with the two-modulator generalized ellipsometer (2-MGE, see the Experimental section), because a commercial CD spectrometer cannot measure the linear birefringence. Figure 1 shows the experimentally normalized Mueller matrices of CLM cell. CLM cell had only linear birefringence and, as a result, *m_23_* and *m_32_*, contributed mostly by LB, and *m_13_* and *m_31_*, contributed by LB’, were larger than other parameters of Mueller matrices. These parameters of Mueller matrix were converted to CD, CB, LD, LB, LD’ and LB’ by the inversion of Jones-Mueller matrices [14,15]. 

**Figure 2:**
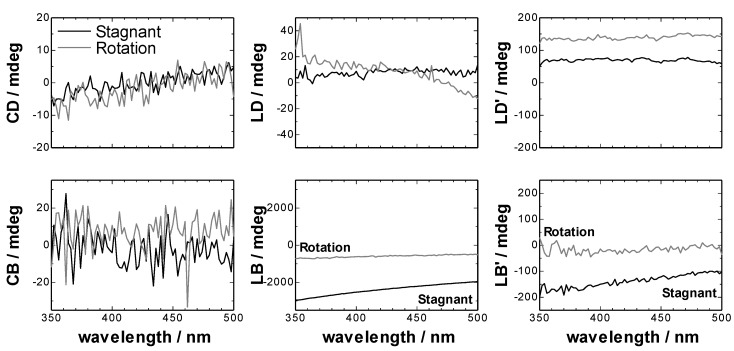
Circular and linear dichroisms and circular and linear birefringences of an empty CLM cell obtained from the inversion of the Mueller-Jones matrices.

The units of CD, CB, LD, LB, LD’ and LB’ were converted from radian to milli-degree, because the commercial CD spectrometer usually use milli-degree as a unit of ellipticity. The converted spectra were shown in Figure 2. 

In Figure 2, it was noted that the intensity of the LB in the stagnant CLM cell was much larger than that of LB’, and the sign of LB was negative, so we can deduce that the slow axis of linear birefringence in the CLM cell directed along the curved wall of the CLM cell. Furthermore, the intensity of LB in the rotating CLM cell became smaller than that in the stagnant CLM cell. It is thought that the LB intensity might be due to the local distortion inside the glass wall of the cell and the heterogeneous value of the LB intensity might be averaged in the high speed rotation of the CLM cell. The decrease of LB intensity of the cell under the high speed rotation is advantageous for the CLM-CD measurement. Finally, it was found that although the linear birefringence of CLM cell varies from one cell to another and also depending on different cell areas, the CLM is appropriate to be used for the optical activity measurements.

### 3.2. Mueller Matrices of Interfacial Heteroaggregates in CLM Cell

**Figure 3:**
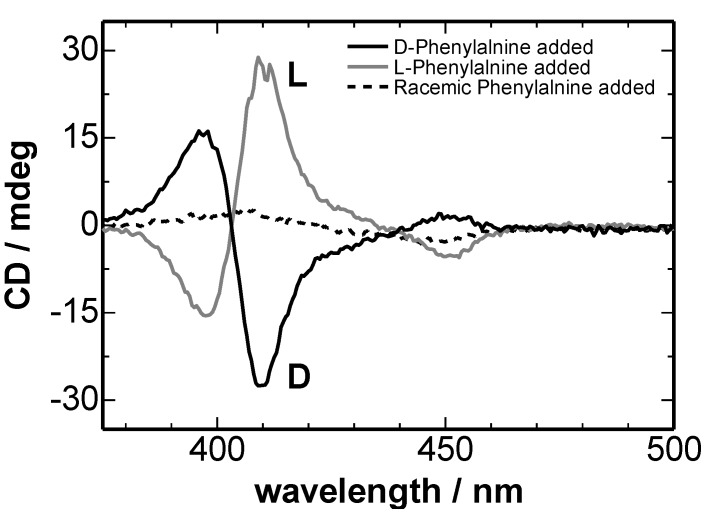
CD spectra of chiral hetero-aggregates at the liquid-liquid interface measured by the commercial CD spectrometer. [TPyP]_org_ = 8.0 × 10^−6^ M, [CuTPPS^4-^]_aq_ = 7.2 × 10^−5^ M, [Phe]_aq_ = 9.6 × 10^−3^ M, [HClO_4_]_aq_ + [NaClO_4_]_aq_ = 0.1 M, pH 2.3, toluene:chloroform = 96:4, v/v, t = 30 min [16].

As a typical sample of the chiral aggregates formed at the liquid-liquid interface, TPyP-CuTPPS hetero-aggregate with l- or d-phenylalanine in toluene-water system was used. The detail of the interfacial formation of the aggregates was reported previously [16]. TPyP-CuTPPS hetero-aggregates in the toluene-water system showed the CD signal corresponding to the chirality of a co-existing amino acid. Figure 3 shows the CD spectra of the TPyP-CuTPPS hetero-aggregates including l- or d- phenylalanine measured by the commercial CD spectrometer. The CD spectra clearly depended on the chirality of the phenylalanine.

**Figure 4:**
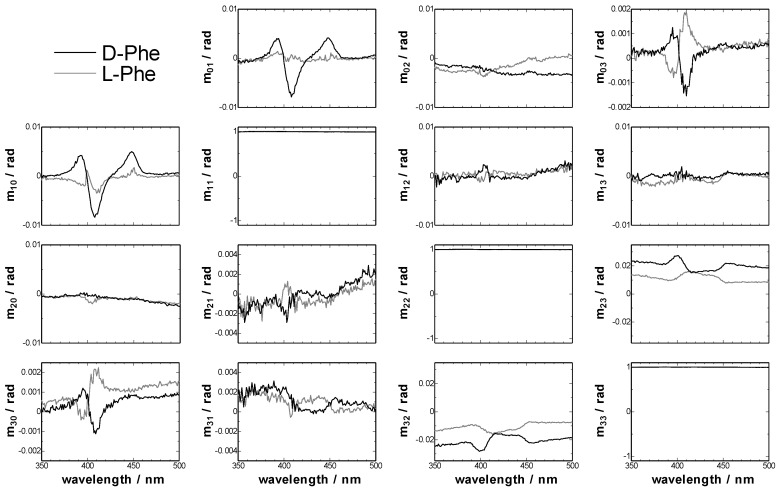
Detail of the experimental normalized Mueller matrices of TPyP-CuTPPS hetero-aggregates in the toluene-water system. [TPyP]_org_ = 8 × 10^−6^ M, [CuTPPS]_aq_ = 1.6 × 10^−4^ M, [Phe]_aq_ = 9.6 × 10^−3^ M, [HClO_4_]_aq_ + [NaClO_4_]_aq_ = 0.1 M, pH 2.3, toluene:chloroform = 96:4, v/v, t = 30 min.

**Figure 5:**
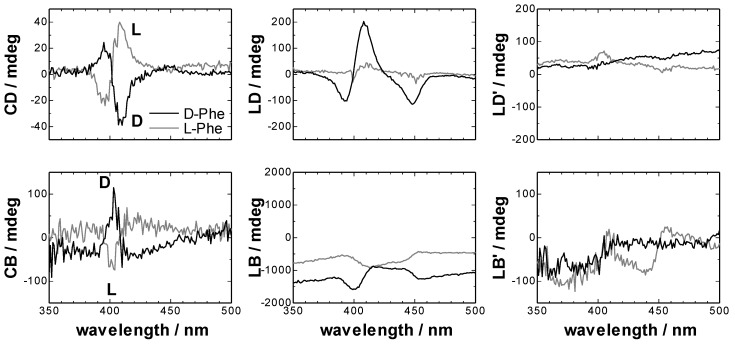
Circular and linear dichroisms and circular and linear birefringences of TPyP-CuTPPS hetero-aggregates obtained from the inversion of the Mueller-Jones matrices. [TPyP]_org_ = 8 × 10^−6^ M, [CuTPPS]_aq_ = 1.6 × 10^−4^ M, [Phe]_aq_ = 9.6 × 10^−3^ M, [HClO_4_]_aq_ + [NaClO_4_]_aq_ = 0.1 M, pH 2.3, toluene:chloroform = 96:4, v/v, t = 30 min.

Figure 4 shows the Mueller matrix elements of TPyP-CuTPPS hetero-aggregates measured by 2-MGE. These parameters were converted to CD, CB, LD, LB, LD’ and LB’ by the same procedure as the empty CLM cell. The results were shown in Figure 5. At first, the Mueller matrices of *m_03_* and *m_30_* in Figure 4, which should include CD signal, showed almost same spectral shapes between them. If *m_03_* and *m_30_* show the opposite sign, these signals should be artifacts and not chiral signals. So, TPyP-CuTPPS hetero-aggregate including d-Phe had a true CD signal, and the value of 1/2(LD’LB – LDLB’) in the element of *m_03_* and *m_30_* was found to be smaller than the CD signal. 

TPyP-CuTPPS hetero-aggregate including l-Phe also had chiral signal by the same reason. In Figure 5, the CD spectrum of TPyP-CuTPPS hetero-aggregates including d-Phe showed the opposite sign against that including l-Phe. CB spectra in Figure 5 were in the Kramers–Kronig consistency with the CD spectra. Thus, it was proved that the chirality of TPyP-CuTPPS hetero-aggregates at liquid-liquid interface could be correctly measured by 2-MGE. But, CD spectra measured by 2-MGE included larger noise than that measured by the commercial CD spectrometer.

### 3.3. CLM-CD Measurements by Conventional CD Spectrometer

CD spectra of TPyP-CuTPPS hetero-aggregates determined by the Mueller matrix measurements were almost same shape with those measured by the commercial CD spectrometer. We will discuss the reason why the CD spectra had same shape.

From the Stokes vector and the Mueller matrices formalisms, Kuroda *et al.* reported that CD signal measured by a conventional CD spectrometer such as Jasco CD polarimeter can be approximated as, [1,2]:

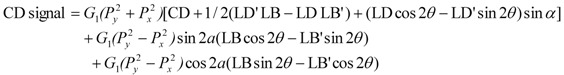
(2)
where *P_x_* and *P_y_* are the transmittance of the photomultiplier along the x and y directions and “*a*” is the azimuthal angle of its optical axis with respect to the y axis, *θ* is the rotation angle of the sample, *α* is the residual static birefringence of the photoelastic modulator (PEM) and *G*_1_ is the apparatus constant related to the sensitivity of the spectrometer at 50 kHz. Here, references [1] and [2] defined the x-axis to be the perpendicular direction and y-axis the horizontal direction. Equation (2) was changed from the original one to make the axis fit to the definition in the present study, *i.e.*, the y-axis to be perpendicular and x-axis the horizontal [1,2].

Because the CD spectrometer was already calibrated, we postulated *G*_1_(*P_y_*^2^ + *P_x_*^2^) = 1. The sin *α* of the CD spectrometer used in the present study was −5.4 × 10^−3^ (at 450 nm), which was determined from the CD measurement of a polarizer, which having only linear dichroism, and Equation (2). The value of sin *α* was the same order to the sin *α* = −7.1 × 10^−3^ (at 250 nm) reported in the literature [17]. In the present study, it was difficult to determine the values of “*a*” and *G*_1_(*P_y_*^2^ - *P_x_*^2^). Shindo *et al.* reported *G*_1_(*P_x_*^2^ - *P_y_*^2^) values were 4.9 × 10^−2^ (at 220 nm), 8.1 × 10^−3^ (at 350 nm), 2.7 × 10^−4^ (at 450 nm) and 2.5 × 10^−4^ (at 560 nm) [1]. These values indicate that the artifacts of CD signal is very small unless the intensities of linear dichroism and linear birefringence are 100 times larger than that of the circular dichroism. 

The term of 1/2(LD’LB – LDLB’) is contained in *m_03_* and *m_30_* of Equation (1). Since the units of LD, LD’, LB and LB’ in Equation (2) are radian, the values of LD, LD’, LB and LB’ are smaller than 1 in any samples. Because the term of 1/2(LD’LB – LDLB’) is the remainder of the product of the values less than 1, the term tends to become small value.

From the comparison of *m_03_* and *m_30_* of Figure 4, the value of 1/2(LD’LB – LDLB’) in Equation (2) was found to be small. In Figure 5, the intensity of the LD of TPyP-CuTPPS hetero-aggregates was 10 times larger than the CD intensity. Therefore, the term of (LD cos 2*θ* – LD’ sin 2*θ*) sin α in Equation (2) should be not so large value. The LB intensity of both the hetero-aggregates and the CLM cell was 100 times larger than the CD intensity. The LB signal of the hetero-aggregates is thought mainly due to the CLM cell. If the value of *G*_1_(*P_y_*^2^ − *P_x_*^2^) of our CD spectrometer around the measurement wavelength is very small like the previous report [1], the terms of the second and the third lines of Equation (2) should also be small. If the value of *G*_1_(*P_y_*^2^ − *P_x_*^2^) of the commercial CD spectrometer in our laboratory was much larger than the reported value of 2.7 × 10^−4^ (at 450 nm) [1], it can be though that the expected artifacts due to the LB of CLM cell was removed by the blank spectrum. Thus, the comparison between Mueller matrix and conventional CD measurements confirmed that the artifacts come from the linear birefringence of CLM cell was negligible in the CLM-CD measurements using a commercial CD spectrometer.

The reason why the CD spectra of TPyP-CuTPPS hetero-aggregates measured by the commercial CD spectrometer was that the orientation of the hetero-aggregates at the interface was very small as found by the small LD and LB. When the interfacial aggregates have large LD and/or LB, it is very difficult to measure the correct CD spectrum of the aggregates by the conventional CD spectrometer. On the other hand, 2-MGE can determine the correct CD spectrum of any aggregates in principle, even of the highly oriented interfacial aggregates. 

## 4. Experimental

### 4.1. Materials

Cu(II)-meso-tetra(4-sulfonatophenyl) porphine (CuTPPS) was purchased from Frontier Scientific, Inc. (USA). 5,10,15,20-tetra(4-pyridyl)-21*H*,23*H*-porphine (TPyP) was purchased from Aldrich (USA). Sodium perchlorate and perchloric acid were purchased from Sigma-Aldrich (USA). Toluene and chloroform were purchased from Scharlab (Spain). d-Phenylalanine (d-Phe) was purchased from Wako (Osaka, Japan). l-Phenylalanine (l-Phe) was purchased from Nacalai Tesque Inc. (Kyoto, Japan) and used as received. Toluene was a poor solvent for TPyP. Therefore, toluene containing 4% chloroform was used as the organic phase solvent. Chloroform was also used to prepare the stock solution of TPyP.

### 4.2. Mueller Matrix Measurement by Two-Modulator Generalized Ellipsometer

The Dual-PEM Mueller matrix polarimeter instrument and the data analysis were essentially the same as those reported previously [18]. The instrument used to measure the Mueller matrices of CLM cell system is a two-modulator generalized ellipsometer (2-MGE) [19,20]. This instrument uses two polarizer-photoelastic modulators (PEM) [19], one as a polarization state generator (PSG) and the other as polarization state analyzer (PSA). On a single configuration, this instrument measures eight independent parameters that correspond to eight different elements of the Mueller matrix. Complete normalized Mueller matrices are measurable by changing the azimuthal orientations of the PSG and PSA.

The Mueller matrix description contains all the information about the polarization properties of a sample, and measurements of the complete Mueller matrix can be used to investigate the optical activity of the sample. However, when many optical polarization effects are simultaneously occurring in the sample, the resulting elements of the experimental Mueller matrix may reflect several effects lumped together, and their interpretation is not straightforward. As a methodology to extract the individual polarization characteristics, the inversion of Mueller-Jones matrices was used in determining CD, CB, LD, LB, LD’ and LB from Mueller matrix [14,15].

**Figure 6:**
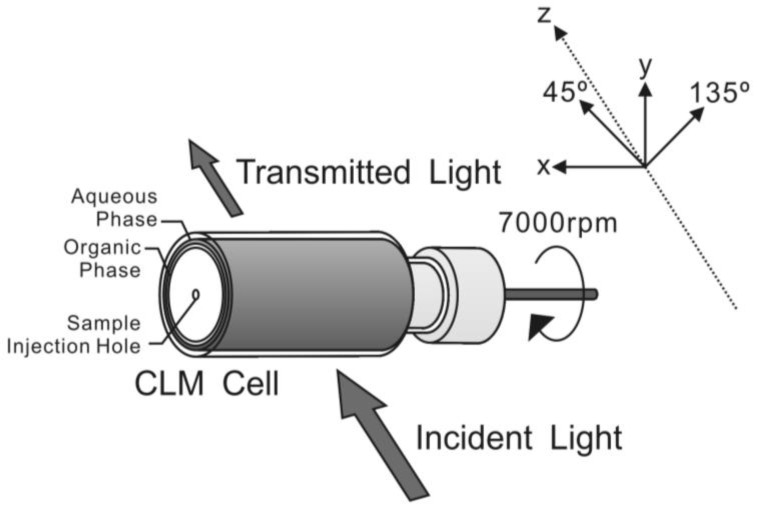
Axes of CLM measurements. x axis was horizontal direction, y axis was the perpendicular direction and z axis was the direction of light propagation. 45° and 135° directions were the directions where 45° and 135° turned clockwise, looked from lightsource, from the x-axis.

The formation of interfacial aggregates at the liquid-liquid interface was directly observed using the centrifugal liquid membrane (CLM) methods combined with 2-MGE method. Figure 6 shows the outline of the CLM method, whose details were reported previously [21]. 

In the CLM measurements, a cylindrical glass cell, having 1.9 cm in diameter and 3.4 cm in length, was placed horizontally, attached by an electric motor in the measurement region of 2-MGE. The procedure for the CLM measurements was essentially the same with that reported previously [16]. The cylindrical cell was rotated at 7,000 rpm by a speed-controlled electric motor (NE-22E, Nakanishi Inc., Japan). At first, 0.480 mL of an aqueous solution of 0.01 M phenylalanine and 0.1 M (H^+^, Na^+^) ClO_4_^−^ (pH 2.3) and 0.480 mL toluene were introduced into the cylindrical cell through a sample injection hole by a microsyringe. Then, 0.020 mL of aqueous solution of CuTPPS and 0.020 mL of a chloroform solution of TPyP were added to initiate the interfacial reaction. The sum of the spectra of the bulk liquid membrane phases, the interface and the glass wall was measured by this method. The calculated values of the thickness of the organic and aqueous phase were 0.26 and 0.25 mm, respectively. The interfacial area between two phases was 20 cm^2^. Figure 6 shows the axes of CLM measurements; x axis was horizontal direction, y axis was the perpendicular direction and z axis was the direction of light propagation. 45° and 135° directions were the directions where 45° and 135° turned clockwise, looked from a light source, in the x-y plane.

### 4.3. Conventional CD Measurement

We used a commercial CD spectrometer of J-820E made by JASCO (Japan). The formation of interfacial aggregates at the liquid-liquid interface was directly observed using the CLM methods. In the CLM measurements, the cylindrical glass cell was set in the sample chamber of the CD spectropolarimeter. The procedure for the CLM measurements was essentially the same with the one described above. At first, a blank spectrum was measured by introducing 0.480 mL of an aqueous solution of 0.01 M phenylalanine and 0.1 M (H^+^, Na^+^) ClO_4_^−^ (pH 2.3) and 0.480 mL toluene with a microsyringe into the cylindrical cell through a sample injection hole. Then, 0.020 mL of aqueous solution of CuTPPS and 0.020 mL of a chloroform solution of TPyP were added to initiate the interfacial reaction.

## 5. Conclusions

We have measured the Mueller matrices of the CLM cell and the aggregates of TPyP-CuTPPS with l- or d-Phe formed at the liquid-liquid interface in the cell. The linear and circular dichroisms and the linear and circular birefringences of the cell and the interfacial aggregates were determined by the inversion of the Mueller-Jones matrices. Thus, the true interfacial chiral signal of the aggregate was obtained by using CLM cell independent of any artifacts. By comparison with the CD spectra measured by a conventional CD spectropolarimeter, it was confirmed that the CLM cell can be used to measure the correct CD of the interfacial aggregate with a commercial CD spectrometer, provided that there are no large LD and LB in the sample. Mueller matrix polarimeter was less sensitive than the commercial CD polarimeter for the measurement of CD spectra, but it can measure all polarization spectra, including the true CD spectra. Thus, the CLM-Mueller matrix method is the general method to measure the polarization spectra of the liquid-liquid interface. This is the first work of the optical activity measurements of chiral molecular complex at the liquid-liquid interface in the CLM cell by the Mueller matrix method. 
